# Fatal hemangioblastoma: a case report and literature review

**DOI:** 10.3389/fonc.2026.1659172

**Published:** 2026-02-04

**Authors:** Wenjie Zhang, Xu Zhang, Chang Ge, Cao Yang, Rui Li, Jingxuan Xu

**Affiliations:** Neuroscience Center, The Second Affiliated Hospital of Xinjiang Medical University, Urumqi, Xinjiang, China

**Keywords:** hemangioblastoma, preoperative embolization, recurrence, spontaneous hemorrhage, surgical treatment

## Abstract

**Background:**

Hemangioblastoma (HB) is a benign tumor of the central nervous system (CNS), typically associated with a favorable prognosis following aggressive surgical resection. Spontaneous rupture and bleeding of these tumors are exceptionally rare, with only a small number of fatal cases reported. The rarity of such cases has impeded reliable epidemiological studies, underscoring the need to investigate the risk factors associated with HB-related mortality.

**Case Report:**

A patient presented with a 12-hour history of headache and rapidly became comatose approximately 30 minutes after admission. Neuroimaging revealed a large, solitary tumor located in the cerebellar vermis, medulla, and C1 segment of the spinal cord, with spontaneous rupture resulting in severe intracerebral hemorrhage. Despite emergency resuscitation and subsequent surgical resection, the patient unfortunately succumbed to their condition. Pathological examination confirmed the diagnosis of hemangioblastoma.

**Result:**

A review of studies published after 2000 identified 21 articles meeting the inclusion criteria, plus one additional case from our hospital, resulting in a total of 30 patients. A summary analysis was conducted on demographic information, age at first diagnosis, time to recurrence, overall survival, maximum tumor diameter, tumor locations (initial and recurrent), tumor texture, presence of VHL, surgical intervention, hemorrhage, cause of death, and embolization status. Missing data were excluded from the statistical analysis. The male-to-female ratio was 18:12, with a mean age at first diagnosis of 40.94 ± 13.44 years. Tumor diameters ranged from 1.6 cm to 5.2 cm, with a median of 3.3 cm. There were 3 cystic tumors, 10 solid tumors, and 2 cystic-solid tumors. Tumor origin sites included the cerebellum (18 cases), medulla (8 cases), and multiple locations (3 cases). Surgical resection was total in 24 cases, subtotal in 1 case, and embolization was performed in 4 cases; 23 cases did not undergo embolization. Seven patients (23%) died within one month, with causes of death including hemorrhage, tumor progression, infection, respiratory failure, and unspecified causes.

**Conclusion:**

Spontaneous rupture and bleeding of hemangioblastomas are extremely rare, and there is currently insufficient evidence to establish clear treatment guidelines. While surgical resection is considered curative, patients with ruptured and bleeding tumors generally have a worse prognosis.

**Abbreviations:**

HB, Hemangioblastoma; VHL, von Hippel–Lindau; CNS, Central nervous system; OS, Overall survival time.

## Introduction

Hemangioblastomas (HB) are benign, highly vascularized neoplasms comprising approximately 2% of intracranial tumors and 3–4% of spinal tumors (1). These lesions predominantly localize to the cerebellum (2), though spinal cord and brainstem involvement occurs in 5% of cases (3). Approximately 20–30% of HB are associated with von Hippel-Lindau (VHL) disease (4). Clinical manifestations primarily stem from secondary intracranial hypertension, encompassing symptoms such as headache, nausea, vomiting, ataxia, and dizziness (3). Intracranial hemangioblastomas generally exhibit favorable prognosis following complete surgical resection, with 5-year survival rates surpassing 90% (5) (6). Hemorrhagic complications are infrequent in this pathology (7). A comprehensive literature review conducted in 2010 identified only 44 documented instances of spontaneous hemorrhage (4). Subsequent research by Ene et al. (2015) reported 53 cases and estimated the incidence of central nervous system hemangioblastomas at 1.41 per million person-years (6) (8). The annual hemorrhage rate among affected individuals is approximately 0.24% (9). Despite this, spontaneous bleeding events remain sparsely documented in global literature.

Current understanding suggests that tumors smaller than 1.5 cm (in size) carry almost no risk of spontaneous bleeding, whereas those larger than 2.3 cm present a significantly increased risk of spontaneous bleeding (7) (9). Ongoing debates persist regarding the mortality rate and prognostic outcomes following spontaneous hemorrhage, as limited short-term mortality data are available (10) (11) (12) (13). Epidemiological studies face challenges due to the scarcity of reported spontaneous hemorrhage cases (8). The present study reports a rare fatal case of spontaneous hemangioblastoma rupture-associated hemorrhage. The tumor exhibited a large, solid morphology, posteriorly situated near the left medulla oblongata. It extended superiorly to the cerebellar vermis and inferiorly to the C1 spinal cord segment. To our knowledge, no prior literature has described a life-threatening event resulting from foramen magnum region hemangioblastoma spontaneous rupture.

## Case report

**Figure 1:**
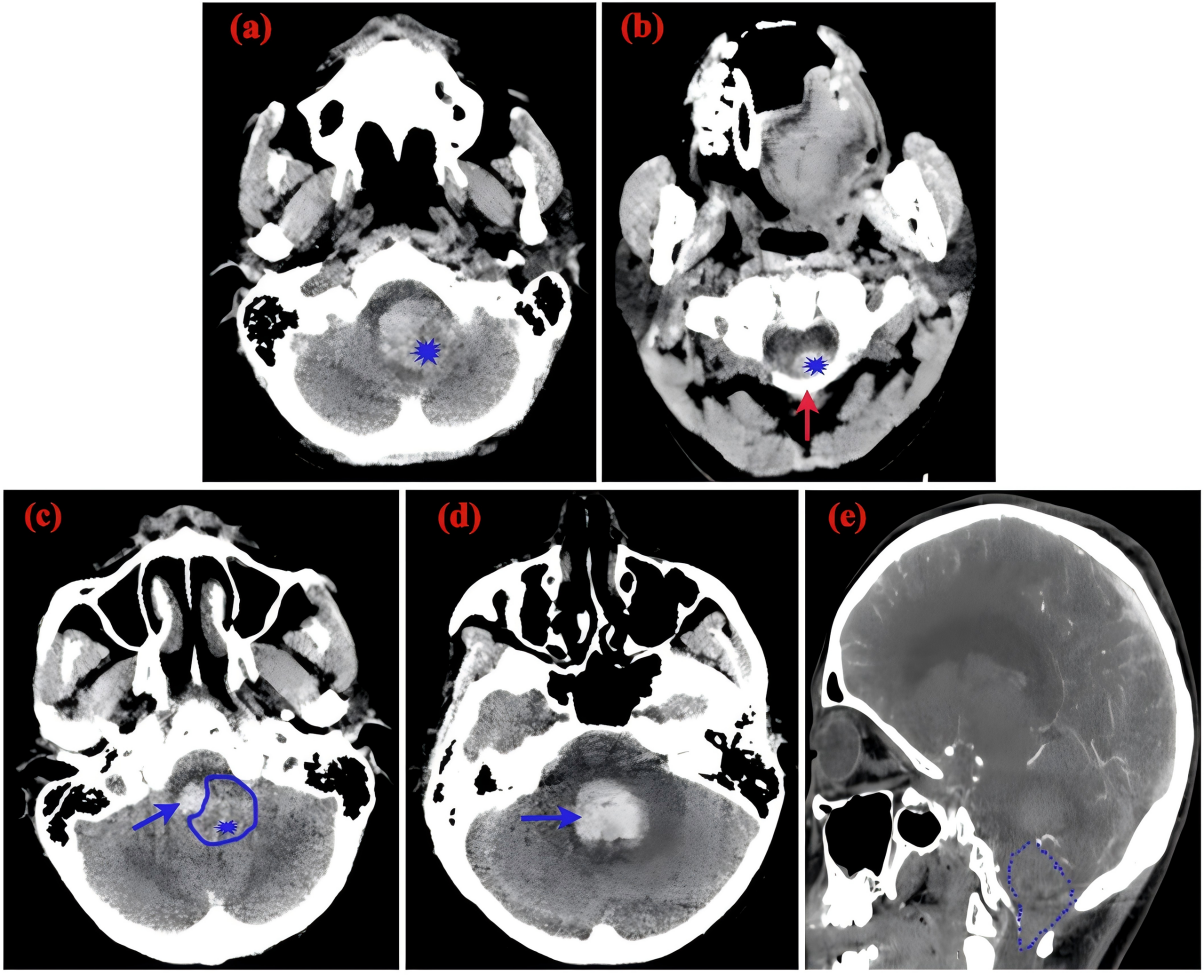
CT scans**(a)** Slightly hyperdense mass in the cerebellar vermis, indicated by the blue asterisk (*), accompanied by a hyperdense hemorrhagic lesion on the right. **(b)** Slightly hyperdense mass extending inferiorly to the cervical spinal cord at the C1 segment (blue asterisk); the red arrow denotes the lamina of the atlas (C1). **(c)** Slightly hyperdense mass in the cerebellar vermis (blue asterisk) and hemorrhage within the fourth ventricle (blue arrow). **(d)** Blue arrow denotes hemorrhage within the fourth ventricle. **(e)** Sagittal view of original CTA image demonstrating the size and location of the tumor (highlighted in blue).

**Figure 2:**
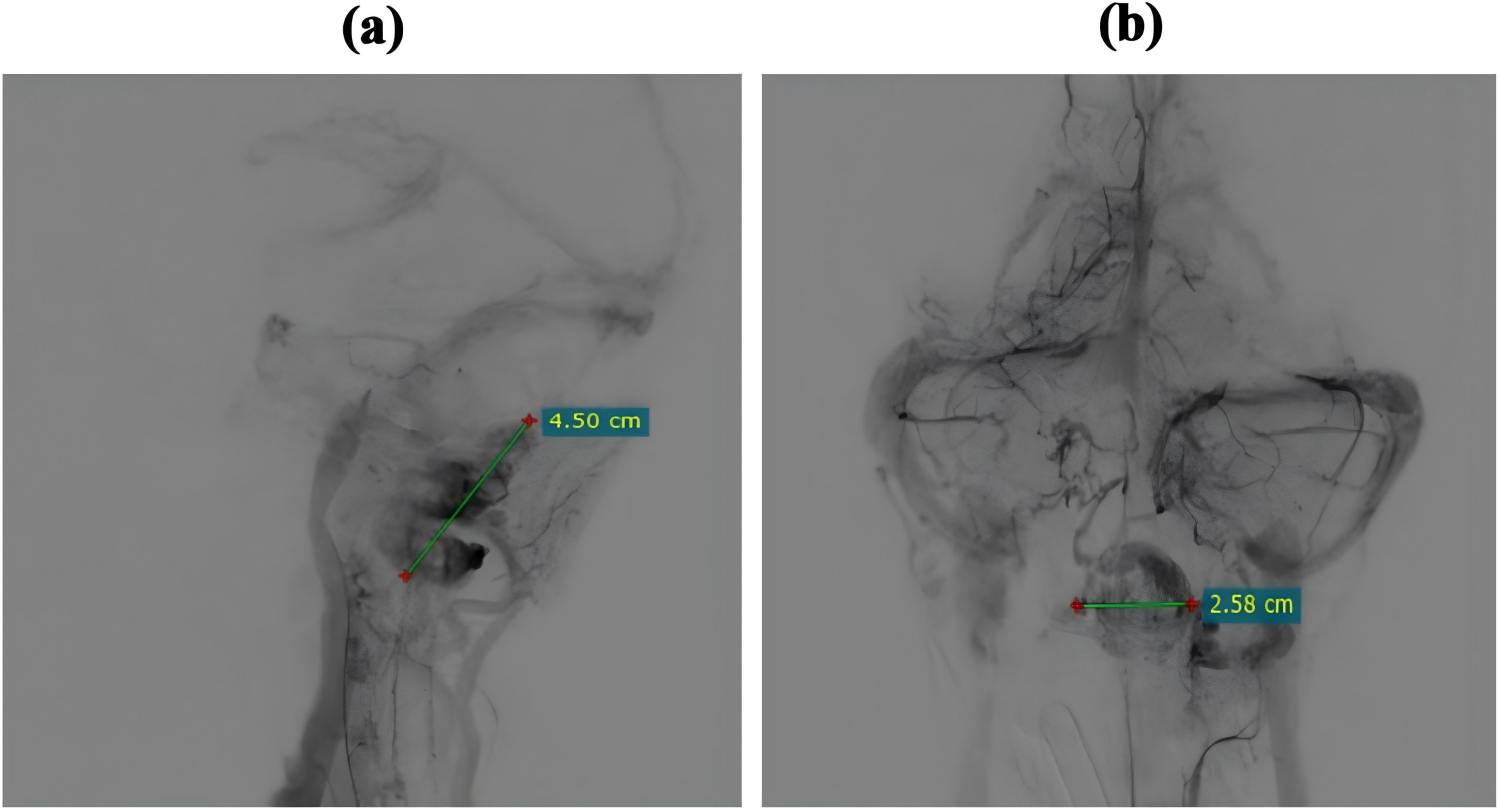
Digital subtraction angiography (DSA) prior to craniotomy. **(a)** Lateral view demonstrating the abnormal vascular lesion with a maximum diameter of 4.5 cm. **(b)** Anteroposterior (AP) view illustrating the transverse diameter of the vascular lesion measuring 2.58 cm.

**Figure 3:**
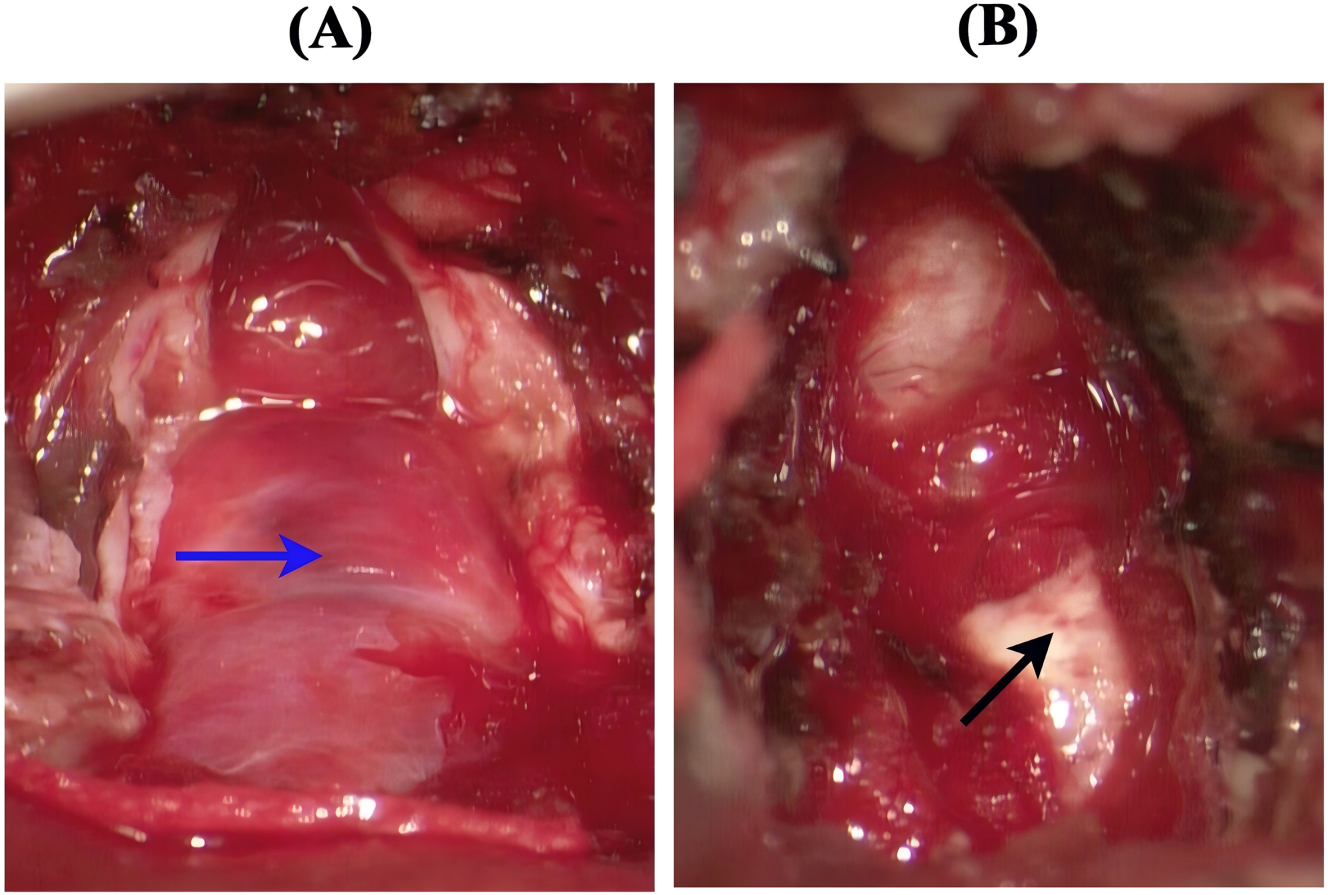
Surgical images. **(A)** The blue arrow indicates a solid mass with an intact capsule, bright red in color and containing abundant blood vessels. **(B)** The black arrow indicates the removal of the tumor from the dorsal side of the medulla, exposing the normal brainstem tissue below.

**Figure 4:**
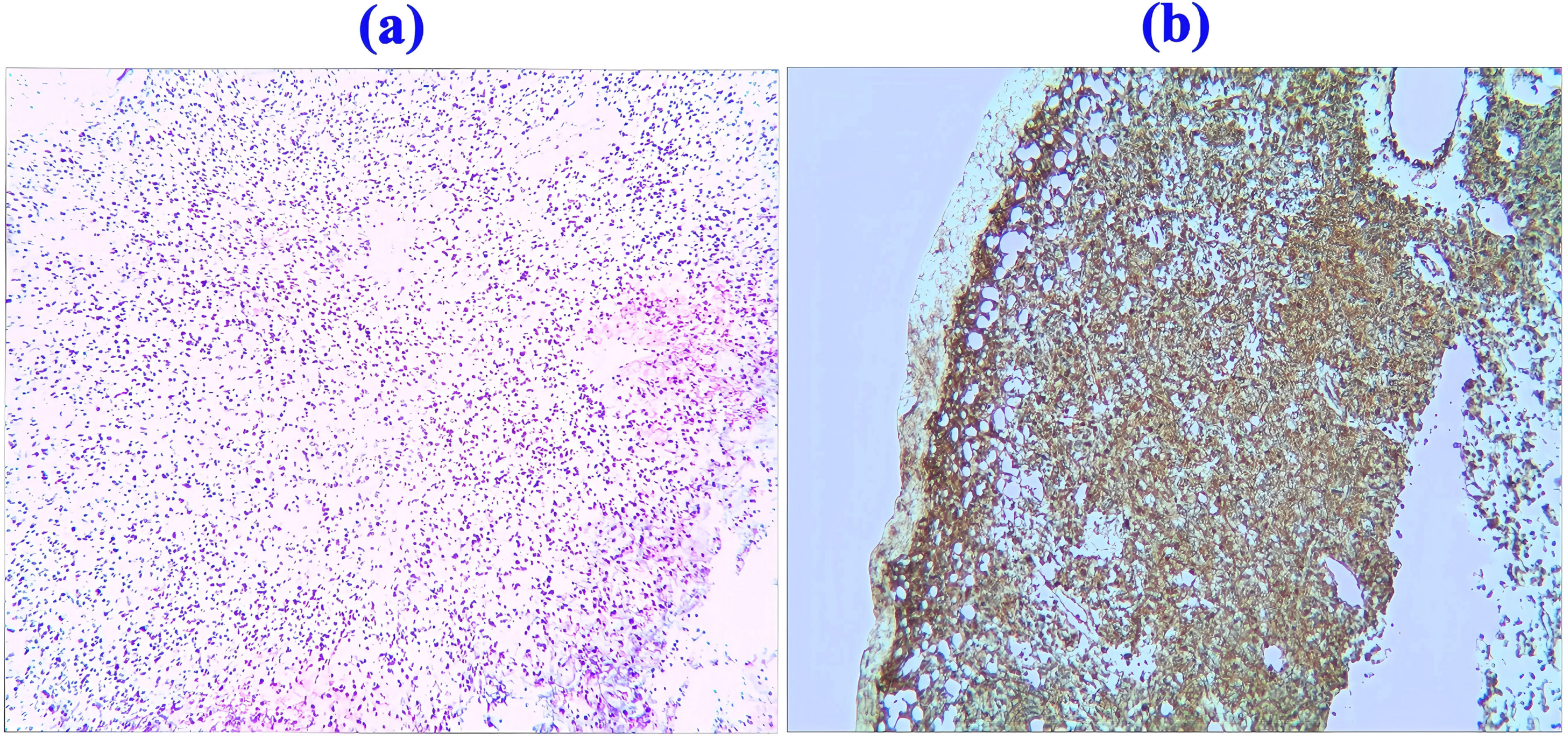
Pathological specimens and immunohistochemistry. **(a)** Histological section stained with hematoxylin and eosin (H&E, ×100) illustrating hemangioblastoma (HB), characterized by abundant stromal cells with clear, vacuolated cytoplasm, focal nuclear atypia, and intermixed capillary vessels. **(b)** Immunohistochemical staining showing positivity for Vimentin (+), CD31 (+), CD34 (+), S-100 (+), focal positivity for NSE (+), and a low proliferative index (Ki-67 ~1%). Negative staining was observed for CD68 (−), GFAP (−), Olig-2 (−), Synaptophysin (−), and Chromogranin A (CgA, −). These findings support the diagnosis of hemangioblastoma.

A 21-year-old female patient presented with an abrupt onset of headache, accompanied by nausea and severe projectile vomiting, persisting for 12 hours. Initial assessment revealed preserved consciousness, but neurological examination documented a progressive exacerbation of headache intensity. Admission to the hospital occurred 12 hours after symptom onset, followed by rapid progression to deep coma within 30 minutes. Physical examination disclosed bilateral 2-mm fixed pupils with sluggish light reflex. Cranial computed tomography (CT) demonstrated a solid tumor and bleeding in the foramen magnum region, with the lesion predominantly on the left side, compressing the medulla. Hemorrhagic extension into the ventricular system and subarachnoid space resulted in fourth ventricle cast formation (**Figure 1**). The clinical diagnosis was established as: occupying lesion in the foramen magnum region; acute obstructive hydrocephalus. Emergency bilateral lateral ventricular puncture and external drainage were performed, with an intraoperative intracranial pressure (ICP) measurement of 200 mm H_2_O. Subsequent digital subtraction angiography (DSA) showed the upper margin extending to the lower third of the cerebellar hemisphere, and the lower margin reaching the inferior border of the posterior arch of the atlas. The lesion measured 4.5 x 2.58 cm in the sagittal plane (**Figure 2**). Emergency resection of the lesion was performed by opening the foramen magnum, exposing the posterior arch of the atlas, and conducting a posterior fossa decompressive craniectomy. During surgery, a firm, gray-yellow, and red solid tumor was observed, with clear boundaries and a surrounding capsule. The tumor was completely resected in segments (**Figure 3**). Pathological examination showed a highly vascularized tumor with abundant capillaries and a rich population of stromal cells. The stromal cells exhibited an epithelial-like appearance with transparent and microvacuolated cytoplasm. Immunohistochemistry revealed positivity for Vimentin (+), CD31 (+), CD34 (+), and focal positivity for NSE (+) (**Figure 4****).**

## Timeline of disease progression

**Figure 5:**
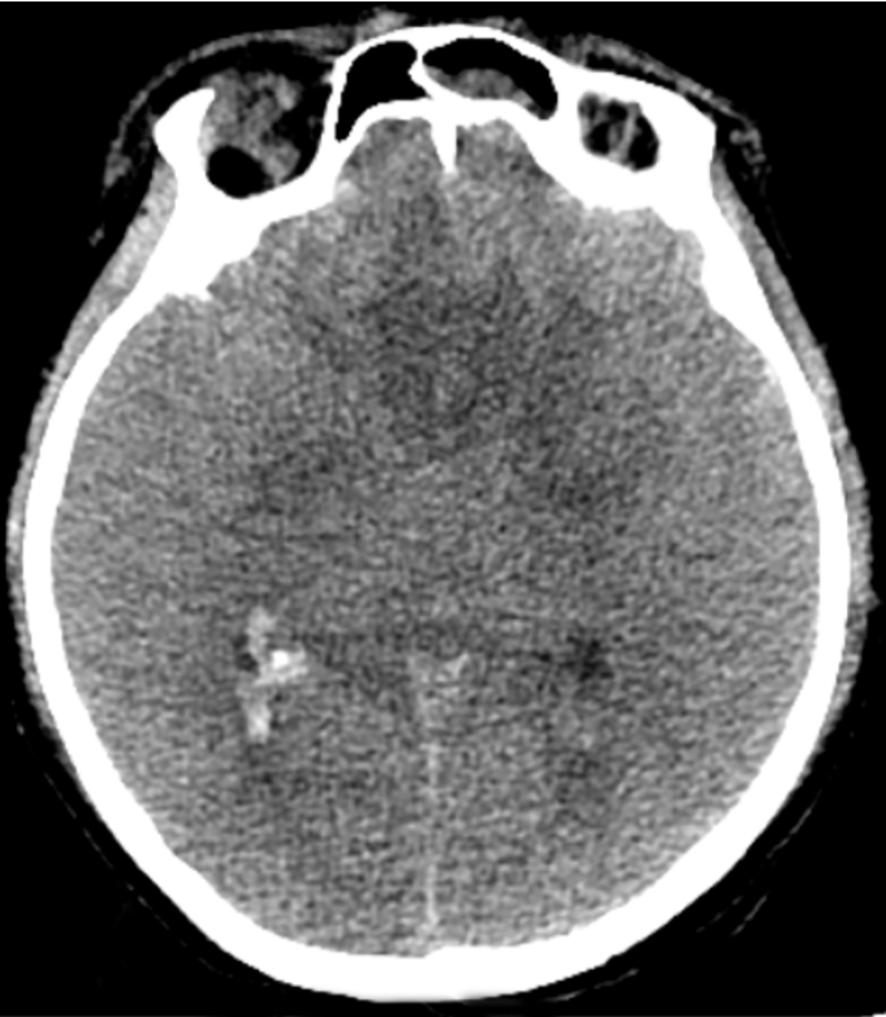
The patient suddenly developed cerebral vasospasm on the second day after surgery, with obvious swelling of the entire brain, disappearance of the cerebral sulci, and low-density lesions in both cerebral hemispheres and the knee of the corpus callosum, indicating cerebral ischemia.

The patient remained in a persistent state of profound coma (Glasgow Coma Scale [GCS] score of 3), accompanied by the absence of spontaneous ventilation, which necessitated endotracheal intubation and mechanical ventilation. On the second postoperative day, serum sodium levels showed a continuous upward trend, reaching a peak of 173.5 mmol/L (normal range: 135–145 mmol/L), requiring bedside hemodialysis. At 04:00 on the third postoperative day, bilateral mydriasis (left: 5 mm, right: 4 mm), accompanied by absent pupillary light reflexes, was observed. Cranial computed tomography (CT) revealed significant cerebral edema with obliteration of sulcal contours and multiple hypodense lesions affecting both cerebral hemispheres and the genu of the corpus callosum. These imaging findings were consistent with cerebral ischemia. Despite the administration of nimodipine, mannitol, edaravone, dipyridamole, and corticosteroids—after the patient's family refused surgical craniectomy decompression—transcranial Doppler (TCD) revealed elevated blood flow velocities in the bilateral middle cerebral, anterior cerebral, and vertebrobasilar arteries, indicating vasospasm. The patient’s clinical deterioration continued, with repeated serum analyses showing neuronal-specific enolase levels exceeding 300 ng/mL (reference range: 0–16.3 ng/mL). Ultimately, the patient succumbed to terminal cerebral edema (**Figure 5****),** refractory intracranial hypertension, and brainstem dysfunction on the eighth postoperative day, with electroencephalography confirming brain death. An autopsy was not performed due to the family's refusal.

## Literature review

### Materials and methods

This descriptive systematic review aims to systematically collect and summarize published case reports and case series on fatal outcomes of hemangioblastoma (HB).

### Eligibility criteria

Literature published after 2000 on fatal hemangioblastoma (HB) patients was reviewed, providing comprehensive information. Studies were considered for inclusion only if they reported data on at least three specific criteria. These included tumor size, age at initial diagnosis, overall survival (and cause of death), primary or recurrence site, the Von Hippel-Lindau (VHL) disease status, and the primary treatment modality (surgery or embolization).

### Search strategy and data extraction

**Figure 6:**
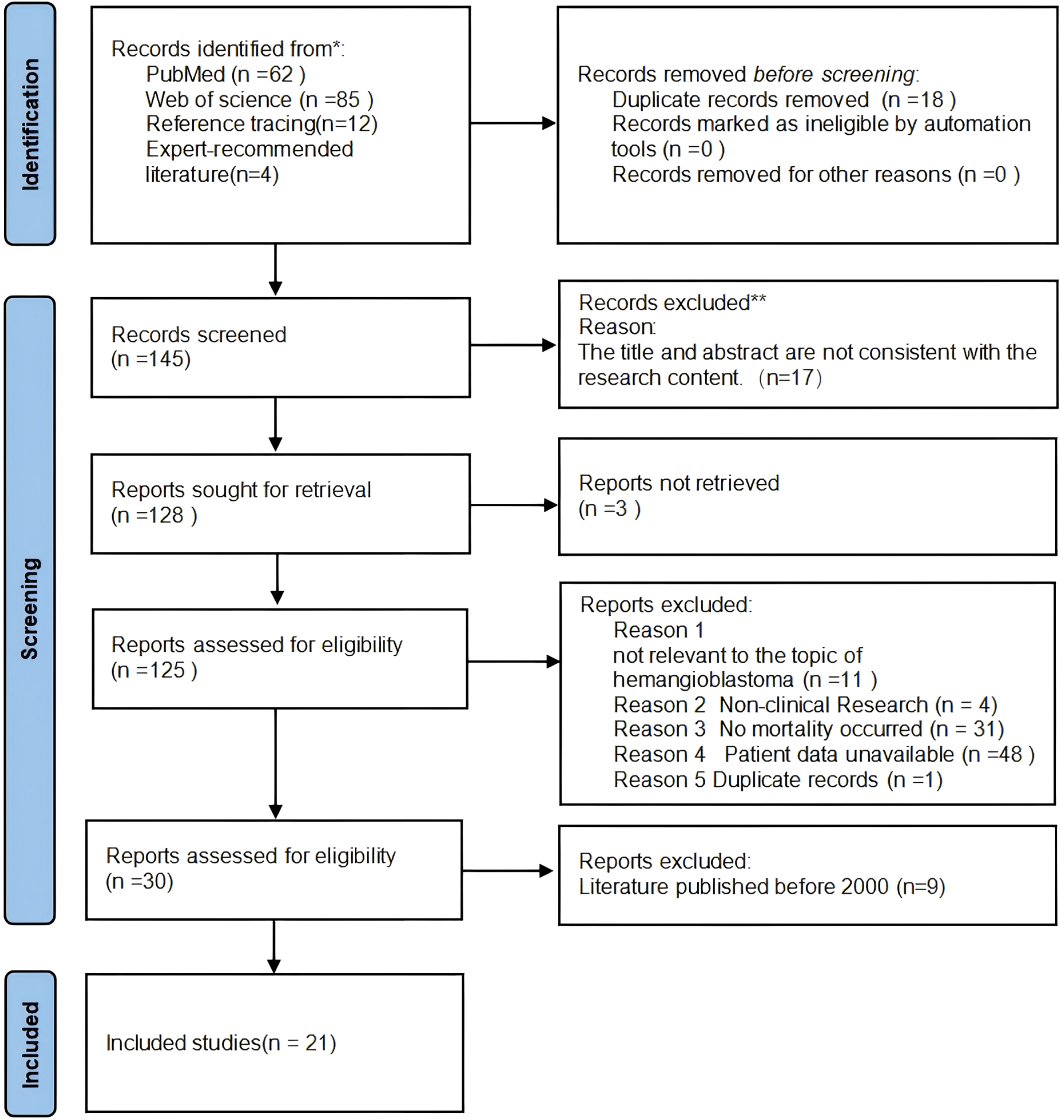
Literature selection flowchart. .

A literature search was conducted using the Web of Science and PubMed databases (without time restrictions). The search terms used were "Hemangioblastoma/mortality", "Hemangioblastoma", "fatal", and "death", with English as the selected language. Animal studies were excluded. Two independent reviewers assessed the article content. Some information was obtained by contacting the authors, and any data that could not be retrieved were recorded as N/A. Data collection and quality assessment were independently performed by two reviewers. In the event of disagreements between the reviewers, an additional reviewer was consulted to reach a consensus. Reference lists were also manually searched to identify additional citations. To ensure comprehensive literature coverage, we also included articles recommended by field experts. The study identification process was conducted in accordance with the PRISMA guidelines, with details provided in the flow diagram (**Figure 6**).

Two reviewers independently assessed the methodological quality of the included studies. As the literature consisted primarily of case reports and small case series, we employed the Joanna Briggs Institute (JBI) critical appraisal checklist for case reports. Disagreements during quality assessment were resolved by consensus or by consultation with a third reviewer. The extracted data and quality assessment results for all included studies are summarized in **Table 1**.

**Table 1:** Characteristics of the included studies.

Time	DOI/PMID	Sex	DA	RT (mons)	OS (mons)	M D(cm)	IS	RS	TT	Surgery		VHL	Bleeding	MC	Embolization
2010	10.1007/s00701-010-0669-7	M	49	N/A	4	2.8	CPA	CPA, Peri-Luschka foramen	Solid	No		No	Yes	Cerebral herniation secondary to post-radiation hemorrhage	No
2025	10.1016/j.esmoop.2025.105109	M	58	N/A	9 days(Post-recurrence therapy)	5.2	N/A	Cerebellum	Cystic-solid	No		Yes	Yes	Belzutifan-associated spontaneous hemorrhage	No
2023	10.1200/PO.23.00066	F	62	N/A	3 days(Post-recurrence therapy)	1.6	Medulla	Medulla	Solid	No		Yes	Yes	Belzutifan-associated spontaneous hemorrhage	N/A
2016	10.1007/s00701-016-2798-0	F	46	68	106	N/A	Cerebellum	Multiple subarachnoid space,spinal cord	Solid	Total		No	No	Death	No
		M	53	136	215	N/A	Cerebellum	Interpeduncular,prepontine,CPA,cisterma magna	Cystic	Total		No	No	Death	No
		M	51	64	69	N/A	Cerebellum	Pons,midbrain,insula,parietal lobe	N/A	Total		No	No	Death	No
2002	10.3171/jns.2002.96.4.0775	F	43	84	102	3.3	Cerebellum	Brain stem, spinal cord	Solid	Total		No	No	Respiratory failure, Aspergillus sepsis	No
		F	47	72	120	N/A	Cerebellum	Cerebellum,medulla,spinal cord	Cystic	Total		No	No	Respiratory failure	No
		M	34	84	121	3.5	Cerebellum	CPA、Cerebrospinal fluid, cervical, thoracic, and lumbar spinal cord, optic chiasm	Cystic-solid	Total		No	No	Stem tumor progression	No
		M	41	96	120	3.5	Cerebellum	Occipital lobe, Brain stem, Cervicobulbar junction, Dorsal aspect of the entire spinal cord	Cystic-solid	Total		No	No	Sudden death	No
2003	PMID: 12699568	M	30	252	264	N/A	Cerebellum	Whole spine	N/A	Total		Yes	No	Death	No
2005	10.1007/s11060-004-2244-7	F	29	264	N/A	N/A	Cerebellum	Brain stem,cerebellum,spinal cord	N/A	Total		No	No	Death	No
2006	10.1016/j.clineuro.2006.12.007	F	23	624	N/A	2.4	Foramen, magnum	Brain stem,spinal cord	Solid	Total		No	No	Death	No
2007	10.1007/s11060-006-9321-z	F	17	168	171	N/A	Cerebellum	Whole spine	N/A	Total		Yes	No	Death	No
2009	10.3346/jkms.2009.24.4.755	M	41	120	132	N/A	Cerebellum	Suprasellar,medulla,spinal cord	Solid	Total		No	No	Death	No
2008	10.3174/ajnr.A1360	M	43	144	168	N/A	Cerebellum	Suprasellar cistern,whole spine	N/A	Total		Yes	No		
2011	10.1007/s00701-010-0827-y	F	46	132	N/A	N/A	Cerebellum	Hypothalamus,temporal lobes,stem, whole spine,	solid	Total		Yes	No	Death	No
2011	10.1007/s11060-010-0244-3	F	28	84	156	N/A	Cerebellum	Cerebellum,brain stem,whole spine	N/A	Total		Yes	No	Death	No
2012	10.1007/s11060-011-0752-9	M	31	60	75	N/A	Cerebellum	Cerebellum,brain stem,multiple spine	N/A	Total		No	No	Death	No
2014	10.4103/2152-7806.142321	F	45	91	120	N/A	Cerebellum	Multiple brain and spine	Cystic	Total		No	No	Death	No
2007	10.3171/jns.2007.106.6.994	M	49	No	Intraoperative	N/A	CPA	No	N/A	No		N/A	Post-embolization hemorrhage	Coma,Death	Yes
		F	25	No	one hour postoperative	N/A	Vermis	No	N/A	No		N/A	Post-embolization hemorrhage	Coma,Death	Yes
		M	71	No	Intraoperative	N/A	CPA	No	N/A	Total		N/A	Post-embolization hemorrhage	Coma,surgery,Death	Yes
2010	10.1136/jnis.2010.004366	M	N/A	No	Within several days postoperative	3.8	Cerebellum	No	N/A	Subtotal Resection		No	No	Intracranial abscess,Hydrocephalus,re-hemorrhage	Yes
2024	10.3390/curroncol31070293	M	56	No	16	2	Cerebellum	No	Cystic-solid	Total		No	No	Recurrence	N/A
2009	10.1007/s10143-008-0166-0	M	26	No	3 days	2.0	Pons, medulla	No	N/A	Total		yes	No	Intracranial abscess, Pneumonia	No
		M	56	No	3	2.0	Medulla	No	N/A	Total		No	No	Pneumonia	No
2010	10.1007/s00701-010-0668-8	M	33	No	16	3.5	Medulla	No	Solid	Total		No	No	Pneumonia,Death	No
2016	10.1007/s00701-016-2834-0	M	33	No	7 days	N/A	Medulla	No	Solid	Total		Yes	No	Pneumonia	No
2025	Present Case	F	21	No	8 days	4.5	Vermis, medulla, Atlas	No	Solid	Total		No	Yes	Intracranial abscess,Stem compression	No

DA, Diagnosis age; RT, Recurrence time (months); OS, Overall survival time (months); MTD, Maximum tumor diameter(cm); IS, Initial site; RS, Recurrence site; TT, Tumor texture; MC, Mortality cause

### Diagnosis

Upon definitive diagnosis of a hemangioblastoma, exclusion of von Hippel-Lindau (VHL) disease is imperative, irrespective of patient age, personal/family history, or tumor localization. Compared to sporadic cases, VHL disease demonstrates heightened familial aggregation and recurrence rates, thereby necessitating rigorous long-term surveillance. This autosomal dominant disorder arises from germline mutations in the VHL tumor suppressor gene (3p25.3) (3) (14), manifesting as multisystem neoplasms, including central nervous system and retinal hemangioblastomas, renal cell carcinoma, bilateral pheochromocytomas, pancreatic cysts, endolymphatic schwannomas, and epididymal cystadenomas (15) (16) (17). In the present case, the tumor exhibited solitary morphology, with no documented family history, absence of retinal vascular anomalies, and unremarkable bilateral renal ultrasonography. Consequently, this lesion was classified as an isolated hemangioblastoma.

However, the final diagnosis of VHL requires genetic testing for germline mutations in peripheral blood leukocytes, with diagnostic accuracy approaching 100% (7, 18). Genetic testing was not performed as consent from the patient's family was not obtained. Recent studies have confirmed that VHL gene alterations can be detected in the vast majority of hemangioblastomas, establishing VHL inactivation as a central event in tumor formation (19). Biallelic VHL inactivation in hemangioblastomas leads to insufficient degradation of HIF-1α, resulting in inappropriate transcription of effectors expressed under hypoxic conditions, such as vascular endothelial growth factor (VEGF) or platelet-derived growth factor B (PDGF-B), which promote tumor formation (20).

Research indicates that approximately 50% of individuals with a mutation in the VHL gene will develop a hemangioblastoma within 30 years of diagnosis (3) (14). Therefore, Suh et al. recommend screening the families of all patients presenting with an isolated hemangioblastoma for VHL mutations to facilitate early identification of at-risk relatives (21).

### Differential diagnosis

Hemangioblastoma must be differentiated from the following conditions: meningioma, metastatic tumors, cystic astrocytoma, medulloblastoma, ependymoma, perivascular tumors, and arteriovenous malformations (AVM). The peak incidence occurs between the ages of 20 and 40. Hemangioblastomas are typically solitary but may be multiple. They can present as large cysts with small nodules or as solid masses. Imaging of hemangioblastomas typically shows large cysts with small nodules, significant and uniform enhancement of the wall nodules, and tortuous blood vessels inside or around the tumor. Pathology reveals a tumor containing numerous foamy cells and abundant capillaries, with positive staining for inhibin α, CD31, and CD34.

### Pathology

Hemangioblastoma comprises two principal components: vascular elements and stromal cells (22). The histological appearance of hemangioblastomas is consistent across various anatomical sites within the central nervous system (16) (23) (24) (25). **Figure 4** shows a dense reticulum network interspersed between the stromal cells and vascular channels. This histological pattern is characteristic of hemangioblastoma (2) (3) (15) (23). In histopathological practice, hemangioblastoma should be differentiated from angiomatous meningioma and metastatic renal cell carcinoma. Useful immunohistochemical markers for this differentiation include positive staining for S100, inhibin-α, and neuron-specific enolase (NSE), and typically negative staining for epithelial markers (2) (3) (15) (25).

### Imaging examination

**Figure 7:**
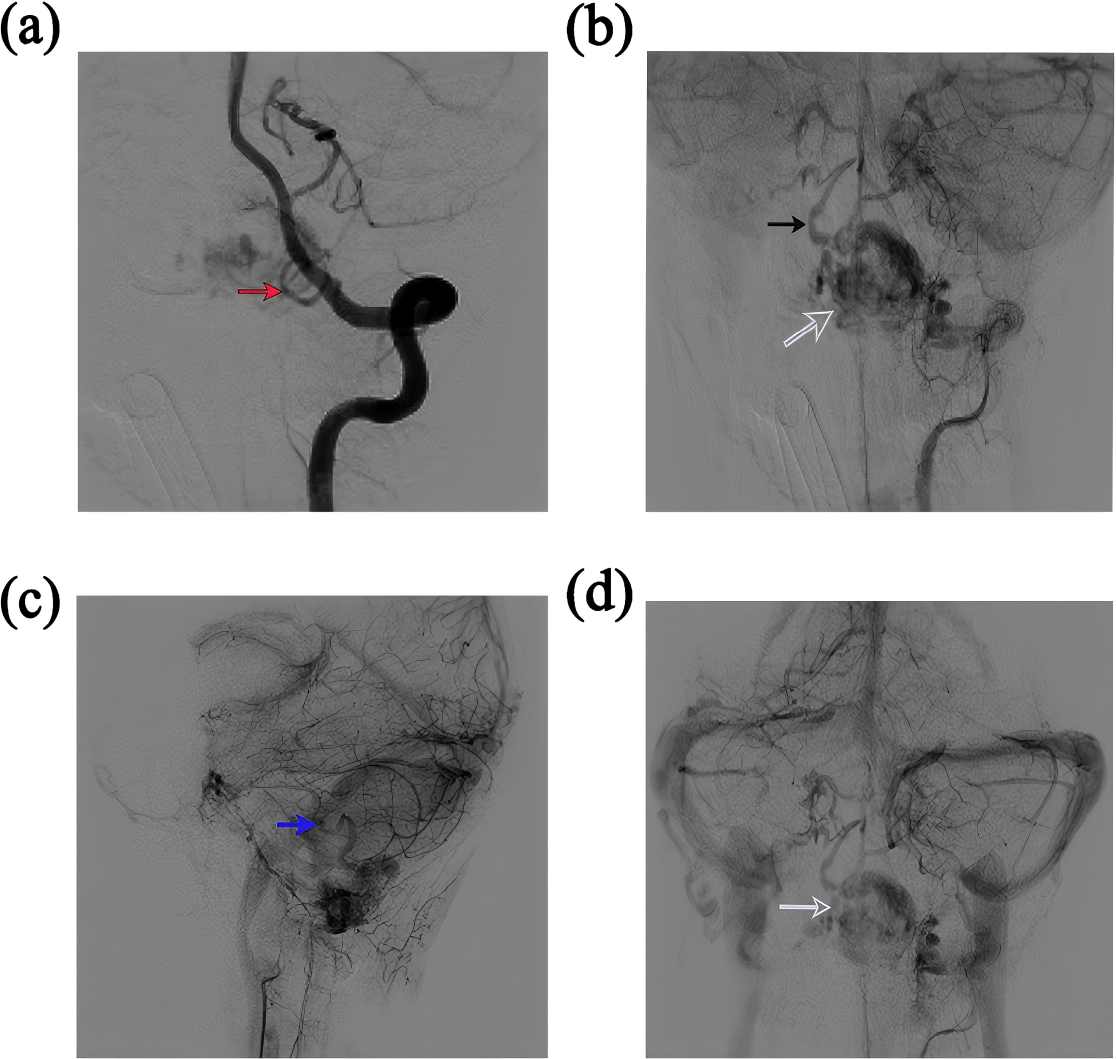
Angiographic characteristics of the lesion. **(a)** The red arrow distinctly indicates an early-phase vascular hemangioma supplied by the posterior spinal artery originating from the vertebral artery. **(b)** The black arrow identifies a prominent draining vein that becomes visible in the late arterial to early capillary phase, a key angiographic feature distinguishing arteriovenous malformations (AVMs) from other solid tumors. White arrows highlight the dense capillary bed characteristic of solid tumors. **(c)** The parenchymal phase demonstrates delayed contrast washout within the tumor tissue. **(d)** During the venous phase, clear staining of the solid tumor components effectively delineates the size and anatomical location of the lesion.

MRI is invaluable for localizing tumors, characterizing their histopathology, and guiding surgical planning (26) (27) (28) (29) (30) (31) (32) (33). However, the prolonged duration of MRI examinations often prevents critically ill patients from undergoing this modality. In this case, the patient was unable to undergo cranial MRI due to life-threatening cerebral hemorrhage. CT remains the first-line diagnostic tool for cerebral hemorrhage but has inferior resolution for solid tumors (29). On CT, cystic components of hemangioblastomas appear as hypodense regions, whereas solid components appear as isodense or mildly hyperdense areas (30). In our case, CT revealed hemorrhage in the medulla oblongata, cerebellar subarachnoid space, occipital cistern, bilateral ventricles, and third/fourth ventricles. Isodense shadows in the posterior medulla oblongata and cerebellar vermis suggested solid tumors. No cystic components were observed, leading to a diagnosis of solid hemangioblastoma. CTA failed to show any significant aneurysms. Digital subtraction angiography (DSA) provides high-resolution visualization of normal and pathologic vascular structures and demonstrates high sensitivity in delineating tumor nodules (3) (12). Hemangioblastomas are highly vascularized, and DSA can reveal the number and distribution of tumor vessels, including feeding arteries and draining veins (31). Such information facilitates preoperative surgical planning, thereby mitigating intraoperative hemorrhage and reducing postoperative morbidity (26) (32) (33) (34). The patient’s DSA showed tumor enhancement during the arterial phase, complete parenchymal phase tumor contour visualization, and prolonged venous phase contrast retention, revealing a tumor volume of 4.5 × 2.58 cm. Angiography further revealed a feeding artery originating from the posterior spinal artery and an abnormally dilated draining vein (**Figure 7**).

However, solid hemangioblastomas often mimic arteriovenous malformations (AVMs) (12), which necessitates careful differentiation. Angiographically, AVMs lack a true capillary bed, whereas hemangioblastomas display microvascular networks that resemble normal capillary beds (4) (7). Such histopathological similarities may impart improved hemodynamic tolerance to hemangioblastomas, leading to a markedly lower bleeding risk compared to AVMs (23) (35).

### Surgical treatment

Surgical treatment remains the gold standard for hemangioblastoma, with complete resection critical for a favorable prognosis (26) (36) (37). The surgical strategy for resecting solid hemangioblastomas differs from that for other tumors and should follow the principles used for arteriovenous malformations. This requires first occluding the feeding arteries while preserving the draining vein until the tumor is fully removed, a technique that prevents tumor collapse or rupture (36) (38). The vascular network of a hemangioblastoma lacks contractile elements. Therefore, the tumor should be removed en bloc whenever possible to avoid uncontrollable hemorrhage (38).

For hemangioblastomas located in the brainstem, surgical resection remains the primary and most effective treatment modality. For hemorrhagic hemangioblastoma (HB), treatment should combine tumor resection with hemorrhage control (4). Mortality significantly increases with intraoperative bleeding (39). Studies have indicated that the mortality rate for surgery in the brainstem region is 25% (12). The brainstem’s complex anatomy poses unique challenges, especially in emergencies. Inadequate surgical planning may lead to brainstem injury, while unavoidable intraoperative bleeding further elevates the risk of poor outcomes (6) (36) (39). Some argue that total resection, rather than piecemeal resection, may reduce the risk of recurrence. Additionally, good prognosis can be achieved even without preoperative embolization (40).

Selective preoperative embolization of feeding arteries, followed by complete tumor excision, is the evidence-supported strategy of choice for mitigating intraoperative bleeding risks and facilitating resection of large solid tumors. Post-embolization reduction in tumor vascularity enhances the likelihood of complete removal while notably decreasing intraoperative hemorrhage (4) (38) (39). Xu demonstrated improved hemorrhage control post-embolization in solid tumors and advocated for preoperative embolization in large solid tumors (9) (38).

However, critics argue that preoperative embolization poses notable risks, including cerebellar infarction, tumor swelling, and enhanced tissue rigidity, which may complicate surgical dissection. Despite these risks, embolization remains clinically safe and effective for brainstem and spinal cord tumors (41) (42). Current literature lacks systematic evidence on the feasibility of preoperative embolization for hemangioblastomas with spontaneous hemorrhage. In our case, the patient presented with severe preoperative symptoms of tumor hemorrhage, which indicated a guarded prognosis. The clinical utility of preoperative embolization in such scenarios remains inconclusive, necessitating further investigation into its potential therapeutic role.

For asymptomatic patients or those unsuitable for craniotomy, long-term observation or radiation therapy may be considered (38) (43). A study showed that stereotactic radiosurgery (SRS) for hemangioblastomas resulted in 7 deaths due to disease progression among 98 patients. A favorable prognosis was observed in cases where the tumor volume was less than 3.2 mL (44).

### Complications

Solid tumors, larger tumor sizes, and lesions near nerve roots are more likely to hemorrhage spontaneously. The primary reasons are as follows:

- Solid tumors possess a denser vascular network than cystic lesions and lack cyst walls for structural support, rendering vessels fragile under conditions like hypertension or trauma (7) (45) (46).
- Large hemangioblastomas (those exceeding 2 cm in diameter) have fragile vasculature, which may increase the risk of spontaneous hemorrhage (9) (47).
- Gläsker et al. attributed the higher bleeding tendency of nerve-root-associated hemangioblastomas to their anatomical relationship with nerve roots, where compression may induce rupture (48).

The present case is consistent with the characteristics previously described. Furthermore, this case represents the largest documented solid hemangioblastoma located in the brainstem.

Subarachnoid hemorrhage secondary to rupture of a brain or spinal cord hemangioblastoma is rare (8) and must be distinguished from aneurysm-related subarachnoid hemorrhage (7). While clinically significant vasospasm frequently complicates aneurysm-related cases (49) (50) (45), this phenomenon has not been documented in hemangioblastoma-associated subarachnoid hemorrhage. Delayed cerebral ischemia resulting from cerebral vasospasm is a leading cause of morbidity and mortality following subarachnoid hemorrhage (51) (52).

The review found that patient mortality was mainly due to hemorrhage, tumor progression, pulmonary infection, and respiratory failure. Hemorrhage occurred spontaneously, following targeted drug therapy, or as a consequence of embolization.

## Discussion

Hemangioblastoma is a benign central nervous system (CNS) tumor that typically has an excellent prognosis following complete surgical resection. Nevertheless, spontaneous rupture and hemorrhage, though rare, can result in severe and potentially life-threatening complications. This study investigates the clinical characteristics, therapeutic challenges, and prognostic factors associated with fatal hemangioblastoma to provide insights for improved clinical management.

The clinical and demographic characteristics of the cohort are presented in **Table 2**. The mean age at initial diagnosis within the study population was 40.94 years. The median maximum tumor diameter was 3.3 cm, with the majority being solid tumors (10 of 15 cases). Tumors were most commonly located in the cerebellum (18 cases) and the medulla (8 cases). Notably, the median tumor diameter in this group exceeded the previously reported threshold (2.3 cm) associated with an increased risk of bleeding, suggesting that tumor volume plays an important role in bleeding and prognosis. Furthermore, the solid composition of these tumors and their location in critical deep regions, such as the posterior fossa and brainstem, increase both treatment complexity and clinical risk.

**Table 2:** Clinical and statistical characteristics.

Clinical characteristics	n (%), mean ± SD, max/min/mdn
Sex	
M	18 (60.00%)
F	12 (40.00%)
DA	40.94 ± 13.44 (Years)
OS	
≤1 month	7 (23.33%)
>1 month	18 (60.00%)
N/A	5 (17.00%)
RT	
Recurrence	17 (%) 146.06 ± 136.64 (Mons.)
N/A	3 (10.00%)
No recurrence	10 (33.33%)
MD	
Available	13 (43.33%) Max:5.2cm, Min:1.6cm, Midian:3.3cm
N/A	17 (56.67%)
IS	
Cerebellum	18 (60%)
Medulla	8 (26.67)
Multiple	3 (10%)
N/A	1 (3.33%)
TT	
Cystic	3 (10%)
Solid	10 (33.33%)
Cystic-Solid	4 (13.33%)
N/A	13 (43.33%)
Surgery	
Yes	Total: 24 (80.00%), Subtotal: 2 (6.67%)
No	5 (17.00%)
VHL	
Yes	9 (30.00%)
No	18 (60.00%)
N/A	3 (10.00%)
Bleeding	
Yes	7 (23.33%)
No	23 (76.67%)
MC	
Hemorrhage	6 (20%)
Tumor progression	3 (10%)
Infection	6 (20%)
Respiratory failure	1 (3.33%)
Unspecified mortality	14 (46.67%)
Embolization	
Yes	4 (13.33%)
No	23 (76.67%)
N/A	3 (10.00%)

Complete surgical resection remains the definitive treatment for hemangioblastoma (53). In our cohort, a complete resection was achieved in 80% of cases (24/30). However, surgical difficulty and perioperative risk increase substantially for ruptured tumors, particularly large, solid tumors in critical locations like the brainstem. The significant intraoperative hemorrhage in our index case highlights the characteristic hypervascularity and complex vascular architecture of these tumors. While preoperative selective arterial embolization can reduce intraoperative bleeding and facilitate resection (54), its optimal timing and safety during acute, life-threatening hemorrhage remain unclear. In our case, rapid clinical deterioration precluded embolization. Future studies should assess whether timely embolization in acute presentations can improve hemorrhage control and outcomes.

In this series, 7 patients (23%) died within one month postoperatively from causes such as re-bleeding, tumor progression, infection, and respiratory failure. In the index case, severe hypernatremia and global cerebral edema developed. This suggests the combined stress of hemorrhage and surgery can disrupt critical regulatory systems, triggering systemic complications. Furthermore, postoperative imaging and transcranial Doppler (TCD) monitoring revealed cerebral vasospasm and subsequent ischemia, a dangerous complication of subarachnoid hemorrhage. Therefore, for hemorrhagic hemangioblastomas, management must extend beyond resection to include meticulous perioperative care of vasospasm, electrolyte balance, intracranial pressure, and systemic status to reduce complication-related mortality.

In summary, while spontaneous rupture and hemorrhage of hemangioblastoma are rare, they are associated with high mortality and require urgent clinical attention. High-risk features may include large tumor size, solid morphology, and location in the posterior fossa or brainstem. Surgically, resection remains definitive, but the approach must balance radical removal with neurological preservation. This decision should be individualized based on tumor location, vascular anatomy, and patient status, and should consider preoperative embolization. Postoperatively, vigilant monitoring and aggressive management of complications such as cerebral vasospasm, elevated intracranial pressure, and endocrine-metabolic disturbances are critical. Future multi-center collaboration and larger case series are needed to better define prognostic factors and establish standardized protocols for the emergency and perioperative management of hemorrhagic hemangioblastomas.
